# Effects of a patient’s name and image on medical knowledge acquisition

**Published:** 2015-12-11

**Authors:** Jesus R. Guajardo, Jean A. Petershack, Julie A. Caplow, John H. Littlefield

**Affiliations:** 1 University of Texas Health Science Center San Antonio; 2 University of Missouri-Columbia

## Abstract

**Purpose:**

To assess whether there are differences in medical students’ (MS) knowledge acquisition after being provided a virtual patient (VP) case summary with a patient’s name and facial picture included compared to no patient’s name or image.

**Method:**

76 MS from four clerkship blocks participated. Blocks one and three (Treatment group) were provided case materials containing the patient’s name and facial picture while blocks two and four (Control group) were provided similar materials without the patient’s name or image. Knowledge acquisition was evaluated with a multiple-choice-question examination (CQA_K).

**Results:**

Treatment group CQA_K scores were 64.6% (block one, *n* = 18) and 76.0% (block three, *n* = 22). Control group scores were 71.7%, (block two, *n* = 17) and 68.4% (block four, *n* = 19). ANOVA F-test among the four block mean scores was not significant; F (3, 72) = 1.68, p = 0.18, η2=0.07. Only 22.2% and 27.3% of the MS from blocks one and three respectively correctly recalled the patient’s name while 16.7% and 40.9% recalled the correct final diagnosis of the patient.

**Conclusions:**

These results suggest that including a patient’s name and facial picture on reading materials may not improve MS knowledge acquisition. Corroborating studies should be performed before applying these results to the design of instructional materials.

## Introduction

There are numerous instructional approaches available to medical education faculty. Virtual patients (VPs) in case-based learning (CBL) are being increasingly used in medical schools and are still considered an innovation by some.^1^ A relatively recent survey of 90 Canadian and US Medical Schools indicated that 94% are using instructional simulation with third-year medical students (MS).^2^ VPs can take many forms, but important elements include an interactive computer simulation of real-life clinical scenarios for the purpose of healthcare and medical training, education and/or assessment.^3^ Recommendations about the importance of authenticity in instructional materials are consistent with the illness script model of learning. The basic idea is that the scripts are created by learners when they acquire experiences with real patients.^4^ Furthermore, the number of actual patients seen with a specific disorder is considered the most important condition in the development of illness scripts.^5^

The Computer-assisted Learning in Pediatrics Program (CLIPP) is a comprehensive Internet-based learning program based on CBL that uses VPs with common clinical conditions typically encountered by third year MS during their pediatric clerkship.^6^ When learning with a *complete* CLIPP^6^ VP case, MS are frequently questioned and provided feedback by the system. It takes MS 25 to 60 minutes to complete a case, depending on the VP condition and case objectives. The *summaries* of the CLIPP^6^ VP cases have no interactivity and provide brief patient data to the reader along with the key medical information that a third-year MS should know about that specific condition or problem.

Development of VPs for medical education is a complex task, as there are many components and variables to consider.^7^ Two potential components are the name of the patient and at least one facial image or a short animation. Recent surveys indicate that MS prefer to have patient images in their case-based learning^8^–^9^ but no formal study has assessed the impact on MS learning. Focus group interviews with MS suggest that the use of VPs improves knowledge acquisition as MS are able to “associate a disease more to a patient than to the textbook”^8^ (p. 3). Botezatu, Hult, and Fors^8^ reported on the importance of visual images as MS from Bogota, Colombia indicated that patients’ images provide “more than a paper case” (p. 6). The authors were “somewhat puzzled by the MS’ emphasis on the importance of the face photo” (p. 7). They also reported that the realism of a case could be conveyed by means of a simple image of the patient’s face and that more advanced media may not be required. An earlier focus group study from Heidelberg, Germany showed similar results to those of Botezatu et al^8^ regarding the use of patients’ images.^9^ The authors recommended presenting an image (or video) at the beginning of the case and also while taking the history to enhance authenticity.

One major question guided the development of this study: does the inclusion of a patient’s name and facial image in a CLIPP^6^ VP case summary improve knowledge acquisition? Therefore the purpose was to assess whether there are differences in MS knowledge acquisition after being provided a virtual patient (VP) case summary with a patient’s name and facial image included compared to no patient’s name or facial image. Our hypothesis was that the inclusion of a patient’s name and facial image in a summary of a CLIPP^6^ VP case would improve knowledge acquisition.

## Methods

**Figure 1:**
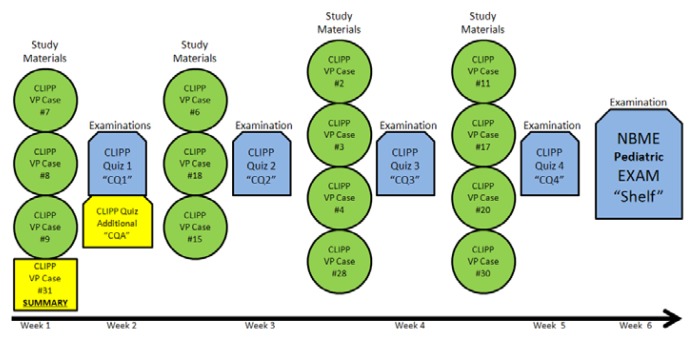
Study interventions (Legend included in test)

The study subjects were third-year MS during their six-week pediatric clerkship rotation. Four successive clerkship blocks of MS were invited to participate in the study which took place during the second half of 2011. MS on the first and third clerkship rotations (hereafter referred to as blocks one and three), were asked to review a summary of a CLIPP^6^ VP case (case 31 Nephrology: Nephrotic Syndrome) along with their routine three other complete CLIPP^6^ VP cases at the beginning of their clerkship rotation (Figure 1).

The case summary was selected (instead of the complete CLIPP^6^ case) as it allowed easier editing of the medical information to keep it similar between Treatment and Control groups and at the request of the course director who thought that adding another complete case would be too much curricular load for the current MS. MS from blocks one and three were exposed to a summary of the CLIPP^6^ VP case containing the name (“*Katie*”) and her facial image (facial picture with no major signs of disease).

This summary showed a single facial image of the patient and provided the medical information adapted to her name which appeared 29 times throughout the text (“*Katie* is 5 years old and …”, “use *Katie’s* history to…”, “*Katie* has had swelling…”, “*Katie’s* case provides…”, “*Katie’s* Differential Diagnosis…”, etc). MS in the second and fourth clerkship rotations (hereafter referred to as blocks two and four) were also provided with the CLIPP^6^ VP case summary containing similar information, but with no facial image or name included; the summary was formatted considering the lack of the patient name and facial image.

All blocks continued receiving the routine and normal curriculum which included 14 CLIPP^6^ cases, four CLIPP^6^ case quizzes (CQ) and the National Board of Medical Examiners (NBME) pediatric exam. For this study, blocks were assessed using the CLIPP^6^ Quiz Additional examination (CQA) which was composed by the Knowledge Component (CQA_K), the Name and Image component (CQA_NI), and two questions referring to *Katie*. The MS were not made aware that part of this study’s evaluation focused on the recall of patient’s names or facial image recognition. The MS enrolled in the study took this study’s test (CQA) along with the first CLIPP^6^ case quiz (CQ1) during their first large group session, one week after they were provided with this study’s case summary.

The CQA_K included 11 MCQs specifically designed for the case used for this study (CLIPP^6^ case # 31). These 11 medical knowledge MCQs related to the field of nephrology were extracted from a large database of questions specifically designed for all the CLIPP^6^ VP cases. Questions from the database have been extensively validated previously and are currently utilized by many medical schools, sometimes as a replacement for the NBME pediatric shelf examination. Recent data (2009–2010) from 777 examinees showed a percent correct mean score of 79.5, a standard deviation of 7.31 and a test reliability of 0.77 for the CLIPP^6^ Test (Berman NB, MedU Co-founder. www.med-u.org. Personal Communication. 7/18/2010).

The CQA_NI included eight MCQs which assessed if the MS were able to correctly recall individuals or final diagnosis of the patients from the three CLIPP^6^ cases of the initial week of the clerkship (cases # 7, 8 and 9. Figure 1). All blocks also had two more questions focusing on matching the facial picture of *Katie* to her name and to identify her final diagnosis. Blocks one and three were scored positively if they correctly answered those two questions, while blocks two and four were scored positively if they indicated that they did not recognize *Katie* and did not know her final diagnosis.

Scores of each of the four weekly CLIPP^6^ quizzes (CQ1, CQ2, CQ3, and CQ4), and of the NBME pediatric shelf examination were obtained. The CQ1, CQ2, CQ3, CQ4 and NBME were not part of this study’s instruments as they were components of the regular and normal pediatric clerkship rotation.

Scores for each block’s performance on the CQA_K were compared to answer the research question of this study. One-way Analysis of variance (ANOVA) was used to compare the percent correct mean scores on the CQA_K and CQA_NI between the four blocks of MS. Effect size is presented as eta squared (η^2^). Fisher’s exact test was used to evaluate the facial image identification and diagnosis recall. Power analysis prior to the study indicated that 20 MS per block would be sufficient to detect group mean percentage differences by F-test at the 0.01 level with power of 93%. These calculations assumed a maximum mean difference of 11.5% in scores with a standard deviation of 8. SPSS 11.5 was the software used for the statistical calculations. Institutional Review Board (IRB) approval was obtained for this study. The MS were provided an information sheet and those who wanted to participate on the study received the appropriate additional materials.

## Results

Of the 108 MS (four blocks) invited to participate in this research study, 76 (70.4%) accepted the invitation. The first block was composed of 18 MS from a pool of 25 (72.0%); the second block had 17 MS out of 27 (63.0%); the third block had 22 MS out of 28 (78.6%); and the fourth block had 19 MS out of 28 (67.9%). The 40 participating MS in blocks one and three were provided with a summary of the CLIPP^6^ VP case that contained the name and facial picture of the patient (Treatment Group). The 36 participating MS in blocks two and four were provided with a summary of the CLIPP^6^ VP case without the name or facial picture of the patient (Control Group).

Treatment Group CQA_K scores were 64.6% (block one, SD=16.8) and 76.0% (block three, SD=16.5) for a combined mean score of 70.9% (SD=17.3). Control Group scores were 71.7% (block two, SD=16.4) and 68.4% (block four, SD=17.0) for a combined mean score of 70.0% (SD=16.5). ANOVA among the four block mean scores showed no statistically significant differences (F (3,72) = 1.68, p = 0.18, η^2^=0.07. Table 1).

Only 22.2% and 27.3 % of the MS from blocks one and three respectively were able to correctly recall the name of *Katie* when they were presented with a question that included her facial picture. Furthermore, only 16.7% of block one and 40.9% of block three MS were able to recall her main final diagnosis. Means for those two questions were 19.4% and 34.1% for blocks one and three respectively. Fisher’s exact tests for the differences between blocks one and three for the two questions were not significant. On the other hand blocks two (88.2%) and four (94.7%) correctly indicated that they did not recognize *Katie* and did not know her final diagnosis.

Blocks one and three *post hoc* analysis of the MS (*n* = 13), who were able to recall Katie’s name and/or final diagnosis versus the MS (*n* = 27) who were not able to, showed no difference on the CQA_K scores (76.2%, SD=16.8 vs. 68.4%, SD=17.4, p=0.18). Analysis of the MS (*n* = 9) who were able to recall both (Katie’s name and final diagnosis) versus the MS (*n* = 31) who recalled none or only one of those variables continued showing no significant differences (77.8%, SD=16.4 vs. 68.9%, SD=17.4, p=0.18).

CQA_NI scores were 47.9% (SD = 31.3), 61.0% (SD=28.9), 55.7% (SD=29.8) and 63.8% (SD=34.3) for blocks one, two, three, and four respectively. ANOVA among the four block mean scores showed no statistically significant differences (F (3,72) = 0.92, *p* = 0.44 See Table 1). The scores on the CQA_NI were higher than the scores on the two questions addressing *Katie* for the Treatment blocks one (19.4%) and three (34.1%). Paired sample *t*-test showed a significant difference (p = 0.003) between the combined all blocks mean CQA_K score (70.5% SD=16.9) when compared to the CQA_NI score (57.1% SD=31.1).

Pearson correlation between all the MS scores (*n* = 76) on the CQA_K (70.5%, SD=16.9) and CQA_NI (57.1%, SD=31.1) examinations was −0.18 (two-tailed significance p=0.13).

Scores for the CQ1, CQ2, CQ3, CQ4, combined CQ scores (CQ1234 mean) and the NBME pediatric component are shown below in Table 1. The mean scores for examinations on the complete CLIPP^6^ cases of the regular curriculum for all blocks combined (CQ1 79.1% SD=13.5, CQ2 85.4% SD=11.6, CQ3 84.3% SD=10.8, CQ4 81.3% SD=14.8) were significantly higher (F(4, 375)=14.3, p < 0.001) than the mean CQA_K score for the four blocks (70.5%, SD=16.9).

## Discussion

This study found that the inclusion of a patient’s name and facial image in a summary of a case-based VP was not associated with an improvement in the scores of MS on multiple-choice examinations. The literature about the inclusion of elements such as patients’ names and images in instructional materials has been primarily descriptive or prescriptive rather than empirical.^8^–^9^ The importance of authenticity in instructional materials (e.g., including patient’s name and picture) aligns with the illness script model of learning^4^–^5^, ^10^ and the stage theory model.^11^

It is possible that the instantiation process of an illness script can be stored in the MS memory without exposure to the image of a VP as text-only materials may produce equivalent results on a multiple-choice examination. Practicing physicians learn in complex clinical contexts where the interaction with the patients is more intricate than just a name and a facial image. Trying to transfer only some of these elements (e.g. patients’ names and facial images) into the instructional materials for MS may not enhance learning compared to text-only materials. In other words, higher fidelity instructional materials and strategies derived from actual practice may not enhance MS learning. This latter idea is supported by a recent literature review of low- versus high-fidelity simulations where conflicting outcomes were found on learning variables.^12^

Some authors believe that overly contextualized knowledge may actually reduce learning.^13^ This belief is supported by concepts associated with information processing theory, such as encoding, which proposes that there is a finite number of units of information that the sensory and working memory can handle.^14^–^16^ Images may convey facts, concepts, processes, procedures and/or principles,^17^ but the patient’s facial image in this study did not convey the presence of disease, therefore it was not reinforcing the medical information provided in the summary. Interestingly and not surprisingly, MS were better at discriminating the lack of previous exposure to a facial picture than associating a name and a diagnosis to a previously seen image.

The name used for patient on this study, “*Katie,*” is somewhat generic and her face did not display obvious signs of disease. It is possible that special or unusual names and patients’ images showing disease may promote better knowledge acquisition than common names and normal facial images. A relationship between empathy and learning has been previously shown,^18^ therefore well thought patient histories may stimulate more empathy and better memory retention. In Medicine, it has been reported that empathy declines if not reinforced in the clinical years^19^ but our study did not focus on assessing empathy.

The mean CQA_K scores were significantly inferior to the scores for examinations (CQs) on the complete CLIPP^6^ cases of the regular curriculum. This could be due to several factors, but two of them may have played the most important roles: time on task and interest in the subject. The complete CLIPP^6^ cases were longer than this study’s summary; consequently MS spent more time on them and that may have helped on memory retention. Additionally, the scores on the related evaluations were part of the pediatric clerkship final evaluation and therefore the MS most likely showed more interest and prepared better for those examinations than for the one associated to this study. The time on task factor may have also influenced the higher scores on name recall and image recognition as all blocks’ CQA_NI scores were higher than the score on the two questions addressing *Katie* for the Treatment blocks one and three. It is quite possible that priming MS for name recall or picture identification may produce much better results than the ones obtained here.

This was a small, one-center only, educational study and therefore had the limitations and possible biases that studies of this type typically have had and most likely will continue to have in similar studies of this nature.^20^ Recommendations on future research based on these limitations involve a multicenter approach with the use of multiple cases (instead of only one), and the use of strategies to improve MS participation and interest on the research materials. Additionally, research may focus also on evaluating knowledge acquisition and other outcomes such as empathy and knowledge retention not only at one week post intervention, but also at one month or even six months in order to assess if patients’ names and images in educational materials contribute to long term retention. Finally, although power calculations were performed a priori to the study, the MS in the study had much higher standard deviations than originally anticipated. The increased standard deviations in the MS blocks diminished the power of this study.

## Conclusions

The addition of names and facial pictures of patients to MS instructional materials does not seem to result in improved retention of key medical information pertaining to the case. Based on the results of this study, it is possible that instructional materials that lack patients’ name and facial pictures may provide an equal opportunity for knowledge acquisition as those that do provide names and images, but without the added expense of obtaining and incorporating those elements to the instructional materials. It appears that those elements may be left as a design choice alone, as they may not impact knowledge acquisition.

## Figures and Tables

**Table 1:** Number of participants and tests scores

	Block1	Block2	Block3	Block4	Mean	F (df 3,72)	*p*
**Number of MS**	18	17	22	19			
**CQA_K Score (SD)**	64.6(16.8)	71.7(16.4)	76.0(16.5)	68.4(17.0)	70.5(16.9)	1.68	0.18
**CQA_NI Score (SD)**	47.9(31.3)	61.0(28.9)	55.7(29.8)	63.8(34.3)	57.1(31.1)	0.92	0.44
**CQ1 (SD)**	75.6(18.5)	80.0(12.2)	80.0(10.7)	80.5(12.2)	79.1(13.5)	0.53	0.66
**CQ2 (SD)**	85.9(12.3)	83.5(12.8)	86.3(9.3)	85.5(12.9)	85.4(11.6)	0.20	0.90
**CQ3 (SD)**	80.2(10.7)	89.6(8.2)	79.1(10.6)	89.4(8.8)	84.3(10.8)	6.60	0.001
**CQ4 (SD)**	72.2(12.4)	83.8(13.7)	82.2(18.0)	86.8(10.1)	81.3(14.8)	3.67	0.02
**CQ1234 mean (SD)**	78.6(7.6)	84.4(6.8)	82.0(7.3)	85.8(7.1)	82.7(7.5)	3.49	0.02
**NBME (SD)**	74.9(9.0)	81.1(11.2)	77.0(7.8)	76.4(5.4)	77.3(8.6)	1.69	0.18

MS: Medical Students. CQA_K; CLIPP^6^ Quiz Additional Knowledge component. CQA_NI: CLIPP^6^ Quiz Additional Name & Image component. CQ: CLIPP^6^ Quiz. SD: Standard Deviation. df: degrees of freedom. NBME: National Board of Medical Examiners. Scores represent the percentage of correct answers on the different examinations.
